# Exploration of motivation to participate in a study of cancer-related cognitive impairment among patients with newly diagnosed aggressive lymphoma: a qualitative sub-study

**DOI:** 10.1007/s00520-021-06527-9

**Published:** 2021-09-08

**Authors:** Priscilla Gates, Haryana Dhillon, Karla Gough, Carlene Wilson, Eliza Hawkes, Lindsay Scudder, Tania Cushion, Meinir Krishnasamy

**Affiliations:** 1 Department of Clinical Haematology, Austin Health, Melbourne, VIC Australia; 2 Department of Nursing, Faculty of Medicine, The University of Melbourne, Dentistry & Health Sciences, Melbourne, VIC Australia; 3 Faculty of Science, School of Psychology, Centre for Medical Psychology & Evidence-Based Decision-Making, The University of Sydney, Sydney, NSW Australia; 4 Department of Health Services Research, Peter MacCallum Cancer Centre, Melbourne, VIC Australia; 5 Olivia Newton-John Cancer Wellness and Research Centre, Austin Health, Melbourne, VIC Australia; 6 School of Psychology and Public Health, LaTrobe University, Melbourne, VIC Australia; 7 Faculty of Medicine, The University of Melbourne, Dentistry & Health Sciences, Melbourne, VIC Australia; 8 Cancer Nursing Research Group, Department of Nursing/Centre for Cancer Research, School of Health Sciences, Faculty of Medicine, Dentistry and Health Sciences, University of Melbourne, The University of Melbourne, Melbourne, VIC Australia; 9 Academic Nursing Unit, Peter MacCallum Cancer Centre, Melbourne, VIC Australia; 10 Research and Education Nursing, Victorian Comprehensive Cancer Centre, Melbourne, VIC Australia

**Keywords:** Participation, Motivation, Cancer-related cognitive impairment, Aggressive lymphoma, Qualitative

## Abstract

**Purpose:**

Cancer-related cognitive impairment (CRCI) is a recognised adverse consequence of cancer and its treatment. This qualitative sub-study was undertaken as part of a larger prospective longitudinal study in which recruitment and retention were very high. The aim was to gain an understanding of participants reasons for ongoing participation, at a time of heightened stress related to a new diagnosis of aggressive lymphoma and the rapid commencement of treatment.

**Methods:**

This qualitative descriptive sub-study included semi-structured interviews with twenty-seven participants. Interviews were recorded and transcribed, and a thematic descriptive approach was used to analyse the data.

**Results:**

Twenty-seven interviews were completed. Four themes described participants’ motivation to consent and continue with the study. These included ease of participation, personal values, self-help and valued additional support. Participants understood the requirements of the study, and data collection occurring during hospital visits was perceived to be convenient. Interviewees confirmed that the study fulfilled desire to “help others”. Although testing was intense and challenging, it provided feedback on current functioning and was described by some as a “welcome distraction” and enjoyable. Finally, interaction with the study nurse was perceived as an additional beneficial oversight and support.

**Conclusion:**

Achieving sustained participation in a prospective study with patients undergoing treatment is facilitated where the logistical demands of data collection are minimised; a clinician from the service is included; the tasks are seen as inherently interesting; and care is taken to provide empathic support throughout.

**Trial registration:**

Australian New Zealand Clinical Trials Registry ACTRN12619001649101

## Introduction

Cancer-related cognitive impairment (CRCI) is a distressing and disabling treatment side effect reported by people undergoing treatment [1–6]. The incidence varies, but studies in people with solid tumours suggest up to 70% receiving chemotherapy self-report some cognitive impairment [2].

Recruitment and retention of participants to longitudinal clinical trials are challenging [7–9], and attrition is often attributed to poor study design [9, 10]. In a review of 18 supportive care oncology trials including 1214 patients, attrition was 44%. Common reasons for dropout were symptom burden (21%), patient preference (15%), hospitalization (10%) and death (6%) [10]. Recommendations to minimise the dropout rate include keeping the study as short as possible, minimising burden on participants and incorporating close monitoring and support for participants [10]. Another study describing the motivations for participation and reasons for adherence in supportive care research reported that participants expressed belief in value to others (96%) or contributing to scientific research (74%). Other responses indicted trust in treating teams (36%) and closer monitoring (28%) [11]. Patients just diagnosed with cancer were less likely to participate due to emotional distress or fear treatment that may be delayed [11, 12]. Research has shown that retention in studies exploring CRCI is challenging [13, 14] with attrition rates in longitudinal cohort studies of cognition in breast and testicular cancer patients ranging from 10 to 33% [15, 16]. The most frequent reasons for study refusal were lack of interest and lack of time [14], and dropout was “feeling overwhelmed” and “losing motivation” [16].

Recruitment and retention to CRCI studies involving neuroimaging can be particularly challenging [13]. A critical component of successful recruitment is having a researcher present the study with confidence and respond to questions in a positive, knowledgeable and reassuring manner. A focus on flexibility in scheduling study assessments to help participants juggle personal responsibilities and medical care and adding neuroimaging onto scheduled scans may assist recruitment and retention [13]. People with cancer may be more receptive to participating in research if a member of the clinical team introduces the study, particularly when pre-treatment baseline assessments are required. Working with clinicians in the early diagnostic work-up is crucial, to ensure that eligible participants are identified early providing a larger window to schedule pre-treatment assessments [13].

## Context of the study

This sub-study was undertaken as part of a larger single-site prospective longitudinal study assessing the feasibility of collecting subjective and objective measures of cognition from people with newly diagnosed, aggressive lymphoma undergoing standard therapy with curative intent [17]. The study was conducted in the haematology department in an acute tertiary hospital in Melbourne, Australia. Referral to psycho-oncology services may have occurred during routine cancer care.

To understand study rationale, it is important to appreciate the situation of potential participants. People were approached and invited to consent to the longitudinal study, sometimes within hours of diagnosis due to the urgency of starting treatment.

Despite this, of the 33 eligible patients, 30 were recruited over 10 months. Participation in the neuroimaging component was optional, 37% were eligible to take part; all agreed (Table1). People were excluded from the neuroimaging sub-study if the diagnostic whole-body positron emission tomography (PET) scan had been completed as no imaging was repeated. Retention and adherence with all assessments were very high at all time points. A single participant withdrew due to disease progression. These data are reported elsewhere.

**Table 1:** Participant characteristics and recruitment

Recruitment rate: total number of patients recruited during time of active recruitment (start of screening to last recruit/consent)		
Recruitment rate (exact Poisson)		
Three patients/month (95% CI: 2.9 to 4.3)		
Consent rate: number consented from number eligible	30	33
Thirty of 33 (91%, 95% CI: 76 to 97%) estimated using the Wilson method		
Reasons for declining participation		
Overwhelmed by potential impact on fertility		
Diagnosis too rushed		
Overwhelmed by diagnosis		
Sex		
Male	16	53.0
Age		
	Median	57
	Range	18–78
Aggressive lymphoma		
DLBCL	20	67
HL	4	13
Other	6	20
Neuroimaging study		
Agreed to participate	11	100
Environment for consenting	30	%
Day oncology	3	10
Inpatient	7	23
Lymphoma clinic	20	67
Where baseline cognitive assessment completed		
Day oncology before chemotherapy	8	26
Day oncology before chemotherapy education	5	17
Day oncology after imaging	6	20
Inpatient	6	20
Lymphoma clinic	3	10
Stand-alone appointment	2	7
Days between diagnosis and consenting		
	Median	2
	IQR	0–7
	Range	0–33
Days between diagnosis and baseline cognitive assessments		
	Median	7
	IQR	2–14
	Range	0–49
Days between diagnosis and commencing chemotherapy		
	Median	12
	IQR	7–17
	Range	0–59

Once recruited, people who had consented to the longitudinal study underwent a comprehensive series of assessments, including neuropsychological testing, self-report questionnaires, blood cell-based inflammatory markers and neuroimaging at three pre-specified time points: pre-treatment, mid-treatment and six to eight weeks post-treatment [17].

Our very high recruitment and retention rate led us to explore what motivated people to take part and stay engaged at such a stressful time. An amendment was submitted to the local HREC to add a participant experience interview, completed after final study assessments. This qualitative sub-study aimed to explore participants’ motivation for sustained participation in a study of CRCI, at the time of a new diagnosis of aggressive lymphoma.

## Method

### Study design

We adopted a qualitative descriptive and exploratory approach. This is particularly relevant where information is required directly from those experiencing the phenomenon under investigation and the aim is to identify the themes that best describe participants’ thoughts and experiences [18, 19].

### Sampling and data collection

Consecutive participants were invited to take part in a semi-structured interview within a week following the final study assessment. Interviews continued to data saturation (three consecutive interviews with no new themes or concepts arising) [20]. Semi-structured interview questions were developed based on key topics of interest through the research team and previous findings [21]. For full interview questions, see supplementary file. Interviews were conducted via telephone at a time convenient to participants. PG completed 24/27 interviews. A trained independent person conducted remaining interviews to reduce potential bias [22]. Interviews were audio-recorded and transcribed for analysis using the framework approach to allow interpretivist understanding of the data [23].

### Data analysis

An interpretivist perspective (interpretive description) guided thematic analysis [19]. Interpretive description allows for investigation of a clinical phenomenon of interest particularly in smaller studies, to capture themes and patterns associated with subjective perceptions to inform clinical understanding [19]. The information provided from each interview was analysed using the framework approach [24] and followed the six phases of analysis recommended by Braun and Clark (2006) [25]: data familiarisation, initial code generation, theme searching, reviewing potential themes, defining and naming themes, co-coding and member checking.

To strengthen trustworthiness, a second coder (LS) reviewed the identified themes and codes in the first four interviews. Additionally, the first six interviews were discussed by study nurse, co-coder and senior researcher. Coding and analysis were discussed with the research team on a fortnightly basis to clarify and interpret emerging themes and sub-themes [22, 26]. Member checking was undertaken to ensure rigour, with the final three participants checking and confirming the key themes to assess trustworthiness of results and to comment on the credibility of themes to the interviewees’ experiences [22, 27].

### Qualitative insights

Twenty-seven interviews were conducted to reach data saturation. All interviews were conducted via telephone, with a median duration of 9 min (range 3 to 22). Participant characteristics are described in the Introduction (see Table 1).

### Themes

**Fig. 1:**
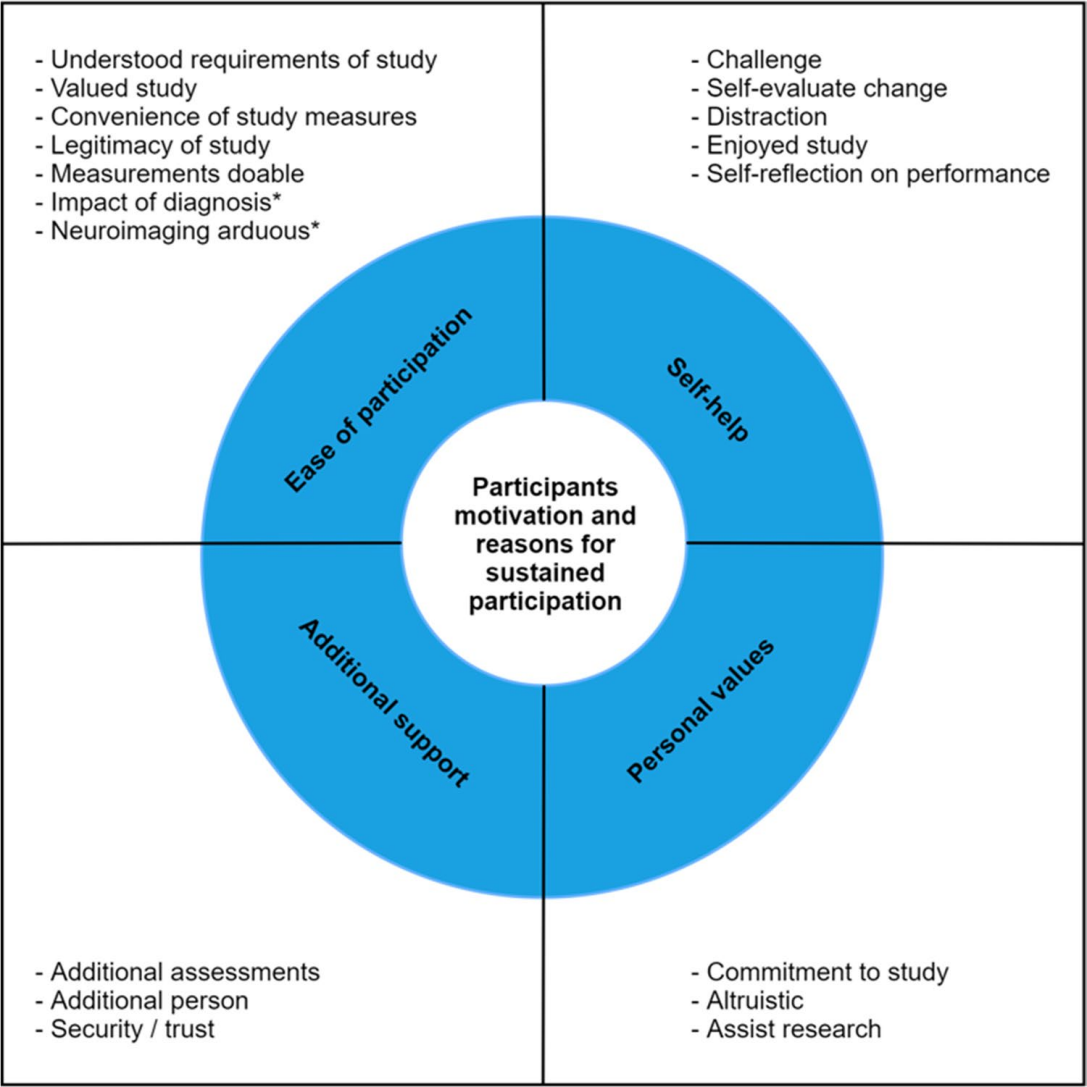
Overview of themes and sub-themes. Note: *potential barrier

Four themes were generated from the data: (1) ease of participation; (2) personal values that impact attitude to participation; (3) desire to engage in self-help; and (4) the appreciation of additional support. These broad themes incorporated a number of sub-themes (see Fig. 1).

#### Theme 1: ease of participation

People spoke of understanding study requirements and perceiving participation as easy and scheduling of assessments as “convenient”. Despite the distress, challenges and stress associated with the cancer diagnosis and concurrent timing of the study invitation, most people approached agreed to participate:

*I was very clear about the information as you presented it very clearly in a way that I could understand, and you also acknowledged that I was in a bit of shock because I had just been diagnosed with Hodgkin lymphoma. (P5)*

However, some participants reflected that, in hindsight, they had felt overwhelmed and stressed when approached. One person mentioned the importance of being given more time to consider study participation”.

*Maybe potentially don’t approach people straight after a big meeting like that. Like for me, it was it was fine, but I could imagine some people may get a bit upset. Maybe mention it, then a phone call or something like that. (P22)*

Many participants commented that the process of taking part in the study was made as easy as possible. They described feeling well informed about study requirements and perceived the study as credible or valued:

*I was kind of excited, a bit privileged, to be a part of the study, to be able to help find out if chemo brain was a thing or not. (P18)*

Organisation of study data collection time points to coincide with planned hospital appointments enabled participation, minimising the study impact on daily life:

*I thought it was well planned as you really made the timing work, and I didn't have to change appointments or chase you at any stage. That was really good because it would have been one thing more on my mind. (P14)*

*All the appointments you tried to work around when I was visiting the hospital anyway. Otherwise, I probably wouldn’t have done it if I'm just coming in for that. (P8)*

By contrast, the neuroimaging sub-study was described by some participants as arduous, anxiety-provoking and demanding. However, it seems that participants valued the study and its importance, which ultimately drove sustained participation (see themes 2 and 3):

*I would have liked more information about how many MRIs and how much time I had to spend at the hospital as I live a long way away. (P24)*

*It's having to lie still for that extra ten or fifteen minutes (PET/CT brain scan), like today they sticky taped my head, which hasn’t happened before. That was frightening, but having cancer was also frightening. (P29)*

Despite this, no participants withdrew from the neuroimaging sub-study or missed a scan.

#### Theme 2: personal values support participation

Participants described wanting to help others as a strong motivator, speaking about a need to help others going through treatment and a desire to improve outcomes:

*I thought if it’s going to help people that have got what I’ve got, it’s all for the good. But if it helps them and helps me, it’s all for the better for everyone. (P5)*

One participant despite feeling confused and stressed at the time that he was approached agreed to do something good for others:

*When they decided that it was lymphoma, there really wasn’t very much time for me, chemo was going to start the next day or that afternoon. It was quite sudden, but I was quite willing to do that, to help others. (P21)*

A few people talked about seeing the study as an opportunity to help others, in the hope it would “send some good” their way:

*I just felt this is something that I can give back. It’s like ying and yang, I felt that if I gave something, some good would come my way. (P23)*

Many described how, having made a commitment to the study, it was important to follow through:

*I'm not a quitter and I didn’t think it was a load. I just wanted to see it through and complete it. (P13)*

One participant mentioned the neuroimaging component as challenging but was committed to completing it:

*There’s no point starting and then halfway saying no. That's just me, anything I undertake, whatever I say I’m going to do, I will always do and fulfil. (P29)*

Another explained that the study was an additional demand on top of an already stressful situation, but despite this, was committed to it:

*I made a commitment and I followed through on that commitment, although there were times when I was anxious and one thing on top of another. It was not so much a burden; it was just another thing to do. Like especially towards the end, I just really didn’t have the energy or motivation if you like. (P15)*

In addition to helping others, participants expressed a desire to contribute to research and build knowledge and understanding about lymphoma and treatment side effects:

*I was really interested to see if we could further the study of my lymphoma and the treatment and its side effects because, I mean, any research is good. (P16)*

One participant described the impact of cancer treatment on another family member as the motivation to take part and complete the study:

*I was pretty on board that after seeing what my sister went through with chemo brain. It certainly looked like a very real thing. I was very interested in the study as well. (P13)*

#### Theme 3: self-help

Here, people talked about how participating gave them an opportunity to help themselves. Undertaking the assessments allowed them to evaluate changes in their cognition as they progressed through treatment:

*The assessments showed me how I was progressing and really to test myself if I have a sort of memory lapse or memory has changed as treatment progressed. (P21)*

In some ways, this gave participants a sense of control, reassurance, and empowerment:

*It is very rewarding to know that you’ve still got a few brain cells there and they’re still functioning. When you go through this you sort of thing, you stop doing a lot because you're not physically capable. When you look at your mental status and you think you’ve gone to jelly, but all those little things in your study, you see it’s all coming back. That was really good. I quite enjoyed it actually. (P14)*

Participants also acknowledged how cognitive testing sometimes resulted in a perception of failure:

*Some of the puzzles that you put me through, like the green, red and blue part, oh my god it did my head in. No, I guess I don't like failure. I think I failed. (P2)*

Although negative experiences were reported, participants generally described enjoying the neuropsychological assessments, perceiving them as a distraction:

*It was a bit of a distraction, to what was going on as well. I thought this may be ok to do just to keep my brain going and not sort of focus on the negative of what else was happening. (P8)*

The neuropsychological assessments in particular were described as enjoyable and stimulating:

*It’s a challenge, a good challenge…… It sort of woke me up again…. it’s really helps you. It opens you up and just jump starts you again. So, when you go back into normal life it helps. (P26)*

#### Theme 4: additional support through participation

Participants valued the additional support and assessments offered during study participation:

*After the second one [treatment] I was a bit worse, and I found the further along I got the worse things got. So a little bit of mental arithmetic and social activity, and an extra person to talk too other than family, and sometimes you need an outsider. (P29)*

One participant described the neuropsychological testing as beneficial.

*The cognition testing helps me because of my age now, just to have this extra testing. (P27)*

Another appreciated the additional neuroimaging, recognising the MRI scan as a test they normally would not have had, and an opportunity for added surveillance:

*I kind of valued having those additional MRIs, to be honest. That wasn’t part of standard of care. (P1)*

The study nurse as an additional team member was highly regarded, perceived as an extra pair of eyes offering surveillance during treatment:

*I was very stressed at the beginning and I really valued there was another person checking in with me. An additional person I would not otherwise have, and I remember thinking that was something I was happy with. (P1)*

This perceived support was enhanced by perceptions of the study nurse’s characteristics:

*You (study nurse) were very respectful. You were very informative, and you laid it out exactly as it turned out to be. (P12)*

Some participants described the positive impact of assessments and interaction with the study nurse had on their thinking, attitude and overall experience:

*I think all the questions stopped me thinking of my own problem, like spending an hour with you I didn’t think of my cancer at all. During your study I’m thinking positive instead of negative, as before I start doing the study with you, I was thinking worst of my sickness. Then I started thinking it’s not the end of the world. (P28)*

Despite the challenging cognitive assessment, the actual appointments were not recalled negatively:

*I was happy when I walked away after every appointment and didn’t feel, you know, harassed or hassled or anything like that. (P12)*

This contributed to creating a safe environment where participants felt a sense of security and trust, resulting in the study assessments being a pleasurable experience:

*This study made my visits here, not a pleasure, but I’d I look forward to it. (P27)*

*I just want to thank you for letting me do this study. Thank you for the distraction before my chemotherapy. Personally, I wanted to thank you, because you’re such a lovely person and was a real pleasure talking to you. (P28)*

## Discussion

This study provides insights regarding motivation and reasons for sustained participation in a study of CRCI, at a time of heightened stress related to a new diagnosis of aggressive lymphoma and the rapid commencement of treatment.

Despite the distressing, challenging and stressful nature of the lymphoma diagnosis, recruiting people at diagnosis was not a significant barrier with 91% of invitees agreeing to participate. It is important to note that participants were approached at the cancer centre by the study nurse which may have contributed to the excellent recruitment rate as the nurse may have been seen as a trustworthy credible member of the team [28]. The study was sometimes introduced by other members of the clinical team, potentially reinforcing legitimacy of the study.

Although the study nurse was a senior nurse within the haematology service, she was not involved in the care of patients with newly diagnosed lymphoma, avoiding conflict of interest. The placement of the study nurse within the clinical service ensured that data collection processes could proceed in a way that minimised demands on participants and maximised gains from the experience. Participants described feeling well-informed, and most indicated a good understanding of the study requirements. They confirmed feelings of trust in the study nurse which supported recruitment and motivation to stay engaged. This is similar to findings reported by Moorcraft et al. (2016) who highlighted the importance of trust in the treating team [11] and Deprez et al.’s (2018) recommendations for maximising recruitment to CRCI studies [13].

Findings from our study contrasted with comments from Moorcraft et al. (2016) who described patients newly diagnosed with cancer, as reluctant to participate in research due to the emotional distress associated with the diagnosis, or a belief that research requirements may delay treatment [11]. Participants in our study reflected on the negative psychological impact of their diagnosis, describing feeling overwhelmed and stressed, with this exacerbated for some by the need to participate in data collection immediately after diagnosis. Consistent with this, a few participants that recommended more time between diagnosis and study commencement would have been ideal. However, this did not impact willingness to participate in our study.

High study engagement was linked by study participants to well-communicated, coordinated and convenient approaches to data collection. A few participants mentioned that they appreciated the planning, as they would probably not have attended the hospital for a study-specific visit. The neuropsychological tests and questionnaires constituting assessments were described as interesting and even useful. However, similar to Deprez et al. (2018), some participants described the neuroimaging sub-study as “challenging” [13]. Despite this, all eligible participants agreed to participate in the neuroimaging sub-study, and none withdrew supporting the view that clear expectations generated high engagement.

Personal values influenced the decision to enrol and continue in the study. Values included “helping” others and “assisting research”, confirming observations from others [11, 29]. Moorcraft et al. (2016) reported that research participants largely agree that they are motivated when “the study results could benefit others” (96%) or “the study would contribute to scientific research” (74%) [11]. Consistent with van Lankveld et al. (2018), participants confirmed “commitment” as a driver of adherence’ additionally, enjoyment and perceived study were influential [29]. However, it is important that clinical staff are cognisant of any study’s demands on participants and their desire to please the team, placing the onus on staff to ensue non-coercive recruitment and follow-up.

Attrition in prospective studies may be minimised when participants perceive a personal benefit. Our participants indicated the study offered an opportunity to evaluate their own cognitive performance and the impact of treatment. Participation was viewed by some as a distraction from treatment, providing a sense of engagement, contribution and purpose. For some participants, distraction was a coping mechanism, buffering negativity that associates with lymphoma and its treatment [30]. It is conceivable that without these positive experiences, attrition would have been higher. Researchers undertaking comparable longitudinal studies need to plan data collection and measures that provide some positive experience for participants and enhance their sense of purpose [10].

It is important to reflect on the relationship between the study nurse (PG) and participants. Although not involved in clinical support at the time of diagnosis, PG was a haematology unit clinical staff member, fully cognisant of the demands of treatment and study requirements. Some participants appreciated the additional support of the study nurse as another person checking on them, and trusting issues would not be missed. This observation confirms reports from Moorcraft et al. (2106) whose participants believed “they would be monitored more closely” [11]. These observations highlight the potential benefit of clinician involvement in data collection [19], strengthening capacity for clinical research among multidisciplinary clinicians, notwithstanding the related ethical concerns of the study nurse-participants relationship grounded in trust but open to participant coercion.

This study has limitations being undertaken as part of a larger longitudinal study of cognition in a single tertiary centre. The number of participants was small and included only one disease type. The interviews were relatively short, although the richness of data suggests that this was not the case. The study nurse coordinating the study conducted most interviews, which may have introduced a bias. In future studies, we recommend a trained independent interviewer conduct interviews to address this concern.

## Conclusion

Our study has highlighted participants’ motivation to participate and stay engaged in a study of CRCI, at the time of diagnosis of aggressive lymphoma and the rapid commencement of treatment. Achieving adherence in a prospective study with patients undergoing treatment was facilitated when logistic demands were minimised; a clinician from the service involved; tasks were seen as interesting; and care was taken to provide empathic support throughout the study. These insights build understanding and inform future studies to shape knowledge addressing cancer and cognition.

## Data Availability

De-identified data supporting the findings of this study are available from the corresponding author upon request.
