# Correction to: Exploration of motivation to participate in a study of cancer-related cognitive impairment among patients with newly diagnosed aggressive lymphoma: a qualitative sub-study

**DOI:** 10.1007/s00520-021-06562-6

**Published:** 2021-09-18

**Authors:** Priscilla Gates, Haryana Dhillon, Karla Gough, Carlene Wilson, Eliza Hawkes, Lindsay Scudder, Tania Cushion, Meinir Krishnasamy

**Affiliations:** 1 Department of Clinical Haematology, Austin Health, Melbourne, VIC Australia; 2 Department of Nursing, Faculty of Medicine, The University of Melbourne, Dentistry & Health Sciences, Melbourne, VIC Australia; 3 Faculty of Science, School of Psychology, Centre for Medical Psychology & Evidence-Based Decision-Making, The University of Sydney, Sydney, NSW Australia; 4 Department of Health Services Research, Peter MacCallum Cancer Centre, Melbourne, VIC Australia; 5 Olivia Newton-John Cancer Wellness and Research Centre, Austin Health, Melbourne, VIC Australia; 6 School of Psychology and Public Health, LaTrobe University, Melbourne, VIC Australia; 7 Faculty of Medicine, The University of Melbourne, Dentistry & Health Sciences, Melbourne, VIC Australia; 8 Cancer Nursing Research Group, Department of Nursing/Centre for Cancer Research, School of Health Sciences, Faculty of Medicine, Dentistry and Health Sciences, University of Melbourne, The University of Melbourne, Melbourne, VIC Australia; 9 Academic Nursing Unit, Peter MacCallum Cancer Centre, Melbourne, VIC Australia; 10 Research and Education Nursing, Victorian Comprehensive Cancer Centre, Melbourne, VIC Australia

**Correction to: Supportive Care in Cancer**

10.1007/s00520-021-06527-9

The article "Exploration of motivation to participate in a study of cancer-related cognitive impairment among patients with newly diagnosed aggressive lymphoma: a qualitative sub-study", written by Gates, P., Dhillon, H., Gough, K., Wilson, C., Hawkes, E., Scudder, L., Cushion, T., Krishnasamy, M., was originally published electronically on the publisher’s internet portal on 08 September 2021 without open access. With the author(s)’ decision to opt for Open Choice the copyright of the article changed on 10 September 2021 to © The Author(s) 2021 and the article is forthwith distributed under a Creative Commons Attribution 4.0 International License, which permits use, sharing, adaptation, distribution and reproduction in any medium or format, as long as you give appropriate credit to the original author(s) and the source, provide a link to the Creative Commons licence, and indicate if changes were made. The images or other third party material in this article are included in the article’s Creative Commons licence, unless indicated otherwise in a credit line to the material. If material is not included in the article’s Creative Commons licence and your intended use is not permitted by statutory regulation or exceeds the permitted use, you will need to obtain permission directly from the copyright holder. To view a copy of this licence, visit http://creativecommons.org/licenses/by/4.0.

The original articles has been corrected.

